# Dataset of white spot disease affected shrimp farmers disaggregated by the variables of farm site, environment, disease history, operational practices, and saline zones

**DOI:** 10.1016/j.dib.2020.105936

**Published:** 2020-06-27

**Authors:** Neaz A. Hasan, Mohammad Mahfujul Haque

**Affiliations:** Department of Aquaculture, Bangladesh Agricultural University, Mymensingh, Bangladesh

**Keywords:** Disaggregated data, Shrimp farming, Risk factors, WSD, Bangladesh

## Abstract

The article presents the summary of a dataset related to the risks factors of white spot disease (WSD) of farmed shrimp (*Penaeus monodon*) in Khulna, Bagerhat and Satkhira districts of Bangladesh. This dataset was developed following two consecutive steps. In the first step, participatory rural appraisal tools were applied to get the conceptual framework for data collection regarding lists of farmers and the variables of the risk factors of WSD. In the second step, sampling of farmers, google featured questionnaire development, and mobile phone-assisted survey were carried out. The total surveyed farms were 233 consisting of 21 and 212 semi-intensive and extensive farms, respectively. The data were collected in the form of continuous, nominal and binary variables disaggregated by saline zones. The dataset contains some basic socio-economic data of shrimp farmers, farm characteristics, environmental attributes and disease history of shrimp farms. The dataset also has GPS coordinates of all the surveyed farms individually which are very useful for spatial analysis. In total, the dataset in MS Excel has 46 variables and attached as the supplementary material with this article.

Specifications Table

**Table:** 

**Subject**	Aquaculture, Aquatic Science, Epidemiology
**Specific subject area**	Aquatic Animal Health Management
**Type of data**	Table
**How data were acquired**	Applying participatory rural appraisal tools; mobile-phone assisted Google featured structured questionnaire survey with shrimp farmers; geographic location of each farm of the respondents
**Data format**	Raw in MS Excel Map for sampled farmers distribution
**Parameters for data collection**	This dataset was obtained from the shrimp farmers following two consecutive steps. Firstly, participatory rural appraisal tools were applied to get the conceptual framework for collecting lists of farmers and the variables associated with the risk factors of WSD. Later, sampling of farmers, google questionnaire development (provided as supplementary file and made available at https://goo.gl/forms/ckG1AIf9xMTxtPpf1), and data collection were undertaken by android mobile phone-assisted survey. Parameters of this dataset belong to the farmers and farm characteristics and management practices of shrimp farms by saline zones.
**Description of data collection**	Total number surveyed farms were 233 consisting of 21 semi-intensive and 212 extensive shrimp farms. The data were collected in the form of continuous, nominal and binary variables disaggregated by saline zones. The dataset contains basic socio-economic data of shrimp farmers, farm characteristics, environmental attributes and disease history of shrimp farms. The dataset also has GPS coordinates of all the farms. In total, the dataset in MS Excel has 46 variables and attached as the supplementary material with this article.
**Data source location**	Institution: Department of Aquaculture, Bangladesh Agricultural University City/Town/Region: Khulna, Bagerhat and Shatkhira districts Country: Bangladesh
**Data accessibility**	Repository name: Mendeley Data identification number: http://dx.doi.org/10.17632/nz96v5spbf.2 Direct URL to data: https://data.mendeley.com/datasets/nz96v5spbf/2
**Related research article**	N.A. Hasan, M.M. Haque, S.J. Hinchliffe, J. Guilder, A sequential assessment of WSD risk factors of shrimp farming in Bangladesh: Looking for a sustainable farming system, Aquaculture. 526 (2020) 735,348. https://doi.org/10.1016/j.aquaculture.2020.735348. [1]

Value of the Data

The dataset of WSD affected 233 shrimp farmers is disaggregated by their farm characteristics and management practices, and by saline zones in southwest Bangladesh which can be used to conduct comparative studies of the changes in shrimp farming on a temporal scale
The key strength of the dataset is that it has GPS coordinates of all the individual farms which researchers and policymakers can use for the establishment of farm traceability that Bangladesh shrimp farms lack severely
The data can be useful for spatial modelling of the impacts of climate change particularly the impact of saline water intrusion on shrimp farming and rural livelihoods
Overall, the data are important for various stakeholders including farmer, policymakers, researchers, scholars, academicians to mitigate the negative impacts of WSD on the entire shrimp farming area of Bangladesh towards sustainable farming

## 1 Data description

The dataset has been built in MS Excel format having two sheets. The first sheet (Dataset) is the main dataset of 46 variables and the second one (DataCoding) is about the coding of different nominal and binary data. The short descriptions of the whole dataset (*N* = 233) are given in the summary Tables 1–3. The data were collected mainly in the form of continuous variables along with some nominal and binary variables. In the summary Tables, continuous variables are presented in average, and nominal and binary variables are in frequency. The basic socio-economic data of shrimp farmers collected includes age, education, farming experiences and farm size are presented in the form of average and frequency (Table 1). The socio-economic data has the potential to disaggregate the whole dataset for comparative analysis within the dataset, and in the future by generating another round of survey data for temporal analysis. Table 2 contains the summary of the dataset for various variables under the domains of farm characteristics, environmental attributes and disease history of shrimp farms. The summary of the dataset related to the data of a range of farm management practices collected from the individual survey site is presented in Table 3. The variables were grouped into five categories in the survey questionnaire (provided as a supplementary file). The key strength of the dataset is that it contains GPS coordinates of all the surveyed farms individually which are very useful for spatial analysis. This dataset will facilitate the researchers to undertake a comparative research on a temporal scale within the same farms, or with neighbouring farms to illustrate the changes of culture practices, and to recommend the way forward towards sustainable shrimp farming in Bangladesh.

**Table 1:** Basic socio-economic characteristics of shrimp farmers.

Variables	Variables type	Variables narration	Average/Frequency
Farmer zone	NV*	Khulna	150
		Bagerhat	26
		Satkhira	57
Farmer age (average years)	CV**	Khulna	42.5
		Bagerhat	41.3
		Satkhira	41.5
Involved with shrimp farming (average years)	CV	Khulna	14.2
		Bagerhat	13.8
		Satkhira	16.6
Farmer education	NV	Primary (1–5)	60
		Junior secondary (6–8)	44
		Secondary (9–10)	60
		Higher secondary (11–12)	41
		Diploma (13–15)	1
		Bachelor's (13–16)	8
		Master's (17–18)	2
		No education	17
Farm size (average in ha)	CV	Khulna	1.28
		Bagerhat	2.86
		Satkhira	2.91

*Nominal Variable; **CV: Continuous Variable.

**Table 2:** Summary of dataset by the variables of site/farm characteristics, environmental aspects and disease history, and by zone.

Variables category	Variables	Variables type	Variables narration	LSZ^1^ (Khulna)	ISZ^2^ (Bagerhat)	HSZ^3^ (Satkhira)
				Average/Frequency		
Site/farm characteristics	Prior land use	NV⁎	Rich or other crops farming: 3	120	23	56
			Wetland or others: 1	30	3	1
	Dominant soil type	NV	Sandy soil: 3	38	3	18
			Loamy soil: 2	93	16	33
			Clay soil: 1	19	7	6
	Average canal depth	CV⁎⁎	Continuous variable	4.52	4.73	3.32
	Average farm depth	CV	Continuous variable	2.7	3.03	1.96
	Culture practice	NV	Extensive: 2	131	24	57
			Semi-intensive: 1	19	2	0
Environmental variable	Temperature	CV	Continuous variable	30.2	27.1	29.3
	pH	CV	Continuous variable	7.8	7.6	7.4
	Salinity	CV	Continuous variable	7.4	10.2	15.9
Disease history	Previous prevalence of WSD	CV	Continuous variable	65.1	57.9	45.4
	Virus detected (current culture)	BV⁎⁎⁎	No: 0	71	5	13
			Yes: 1	79	21	44

1 LSZ: Low Saline Zone.

2 ISZ: Intermediate Saline Zone.

3 HSZ: High Saline Zone.

⁎ NV: Nominal Variable.

⁎⁎ CV: Continuous Variable.

⁎⁎⁎ BV: Binary Variable.

**Table 3:** Summary of the dataset by different management variables of shrimp farming practices, and by zone.

Variables category	Variables	Variables type	Variables narration	LSZ^1^ (Khulna)	ISZ^2^ (Bagerhat)	HSZ^3^ (Satkhira)
				Average/Frequency		
Management variables (Site/farm management)	Farm operated by owner	BV⁎⁎⁎	No: 0	26	5	5
			Yes: 1	124	21	52
	Use of fertilizer	NV⁎	No: 4	59	9	8
			Inorganic: 3	62	9	41
			Organic: 2	10	8	1
			Mixed – inorganic and organic: 1	19	0	7
	Chemicals use (pond preparation)	NV	Chemical treatments: 3	24	7	7
			Therapeutic treatments: 1	126	19	50
	Chemicals use (water treatment)	NV	Chemical treatments: 3	60	11	11
			Therapeutic treatments: 1	90	15	46
	Use of aerator	BV	No: 0	132	22	56
			Yes: 1	18	4	1
	Gher drying after harvest	BV	No: 0	6	1	0
			Yes: 1	144	25	57
	Sludge removal method	NV	No: 5	18	3	13
			Flushing, deposit sludge on farm: 3	62	9	24
			Flushing, deposit sludge on and off farm: 2	48	7	17
			Flushing, deposit sludge off farm: 1	22	7	3
	Sludge removal interval	NV	Never: 1	18	3	13
			1 year: 2	102	17	39
			≥2 year: 3	30	6	5
Management variables (Site/farm management)	Maintain and repair dikes	NV	No repaired dikes or repair with the pond bottom soil of other farms: 4	7	1	1
			Repaired dikes with the pond bottom soil of farm itself: 2	134	23	56
			Repaired dikes with the soil from fallow land: 1	9	2	0
	Period of fallow	CV⁎⁎	Continuous variable	55.56	57.3	45
Management variables (Water management)	Water source (direct natural)	NV	Rain water: 3	6	1	0
			Boring water: 2	21	0	3
			Direct from sea or river/tidal flow: 1	56	11	10
			If not direct natural: 0	67	14	44
	Water source (indirect natural)	NV	Water coming via other shrimp farms: 4	28	9	10
			Canal from sea/river: 2	20	3	34
			Treated water: 1	19	2	0
			If not indirect natural: 0	83	12	13
	Water coming via other farms	BV	No: 0	122	17	47
			Yes: 1	28	9	10
	Reservoir	BV	No: 0	135	25	57
			Yes: 1	15	1	0
	Frequency of water exchange	NV	≤ 7 – 28 days: 4	43	17	26
			29 – 42 days: 3	49	3	7
			> 42 days: 2	14	1	6
			No exchange: 1	44	5	18
	Same passes for inlet/outlet	BV	No: 0	65	8	7
			Yes: 1	85	18	50
Management variables (Culture management)	Culture method	NV	Monoculture: 4	20	4	1
			Polyculture (shrimp with prawn): 3	34	10	6
			Polyculture (shrimp with fish): 1	96	12	50
	Source of PL	NV	Mixed source or non-registered private hatchery: 3	19	1	7
			Registered private hatchery: 2	99	17	48
			Wild: 1	32	8	2
	Stocking density	CV	Continuous variable	229.7	208.7	257.1
	Stocking age	CV	Continuous variable	13.8	22.9	16
	Quality of PL	NV	Low: 3	9	2	1
			Medium: 2	115	23	56
			High: 1	26	1	0
	Crop rotation	BV	No: 0	82	11	26
			Yes: 1	68	15	31
Management variables (Feed management)	Types of feed use	NV	Live food: 5	12	1	20
			Homemade pellet feed: 4	25	9	7
			Mixed use of homemade and commercial pellet feed: 3	40	12	8
			Formulated commercial pellet feed: 2	50	4	3
			No: 1	23	0	19
	Use of feed additives	BV	No: 0	94	6	43
			Yes: 1	56	20	14
Management variables (Biosecurity management)	Bird scare net	BV	No: 0	57	25	57
			Yes: 1	0	1	0
	Crab fence	BV	No: 0	57	24	57
			Yes: 1	0	2	0
	Footbath	BV	No: 0	57	24	57
			Yes: 1	0	2	0
	Limited access	BV	No: 0	54	23	54
			Yes: 1	3	3	3
	Same equipment for the whole farm	BV	No: 0	0	2	0
			Yes: 1	57	24	57

1 LSZ: Low Saline Zone.

2 ISZ: Intermediate Saline Zone.

3 HSZ: High Saline Zone.

⁎ NV: Nominal Variable.

⁎⁎ CV: Continuous Variable.

⁎⁎⁎ BV: Binary Variable.

## 2 Experimental design, materials, and methods

This dataset was developed following two consecutive steps. In the first step, participatory rural appraisal tools such as key informant interview (KII), focus group discussion (FGD) and field observations were conducted to get the conceptual framework for generating lists of farmers and the variables associated with the risk factors of white spot disease (WSD). In the second step, sampling of farmers, google featured questionnaire development, and data collection were carried out by android mobile phone-assisted survey. In the beginning, through extensive literature review particularly reviewing the statistical report published by Fisheries Resource Survey System (FRSS) of the Department of Fisheries (DoF), the major shrimp producing sites were selected in Khulna, Satkhira and Bagerhat districts of Bangladesh (Table 4). Shrimp farming in Bangladesh is characterized by a large number of small farms (over 200,000 farms registered by DoF), weak traceability, extensive farming practices, mass mortality due to WSD almost every year, and vulnerable to climate change [2], [3], [4], [5], [6], [7], [8], [9].

**Table 4:** Top shrimp producing districts in Bangladesh by volume of production (adapted from [2]).

District	Shrimp production (MT)	% of total production
Khulna	56,043.48	22.03
Bagerhat	64,607.96	25.4
Satkhira	64,875.91	25.5
Jessore	37,643.13	14.8
Cox's Bazar	22,944.93	9.02

**Fig. 1:**
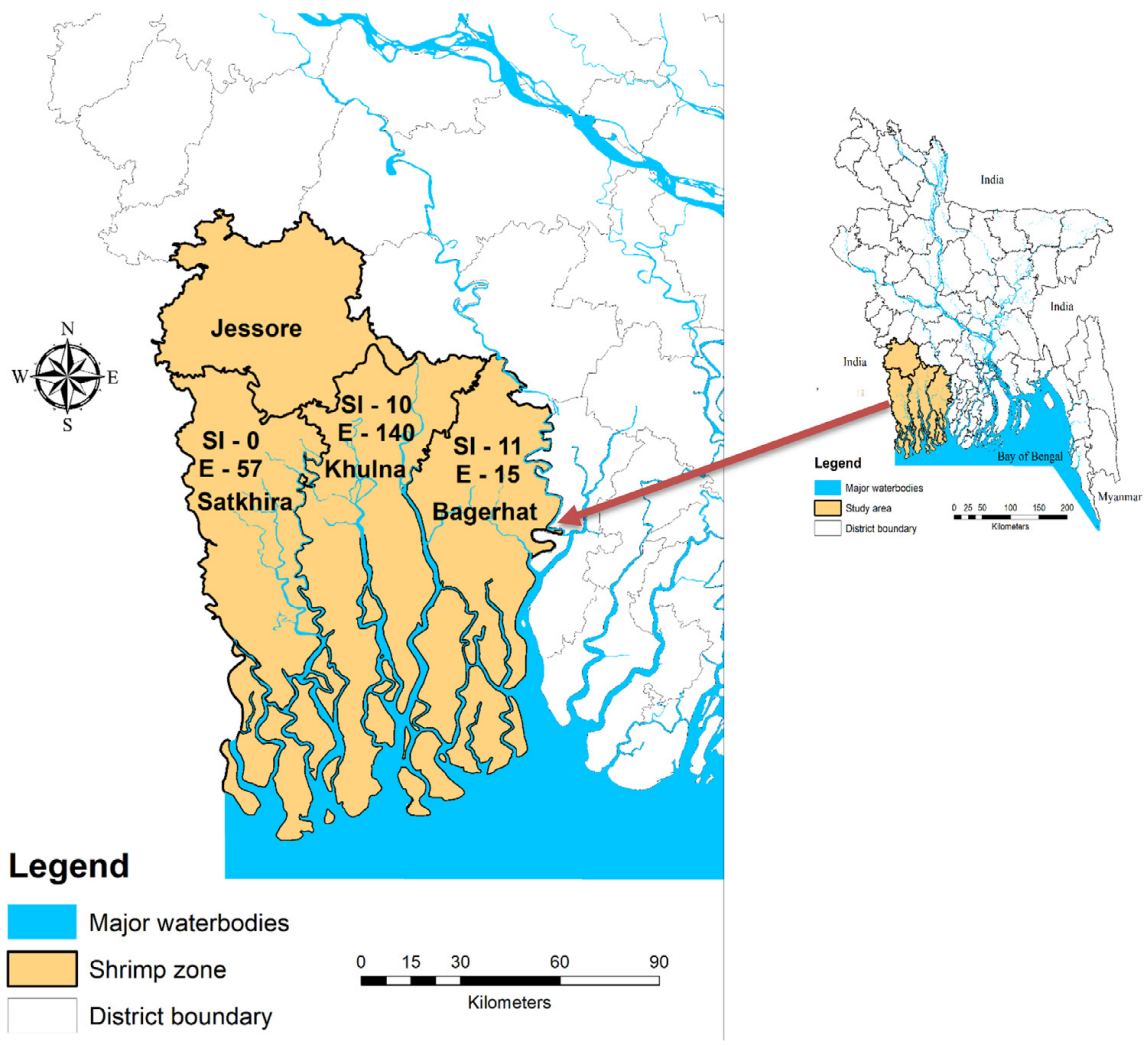
Map of Bangladesh showing the distribution of sampled shrimp farmers (SI=semi-intensive; E=extensive) in data collection area.

These sites are collectively known as the ‘*shrimp zone*’ consisting of high saline, intermediate saline and low saline areas from where comprehensive lists of WSD experienced shrimp farmers were collected from the key informant, local Upazilas (sub-districts) Fisheries Officers of the DoF. The list of shrimp farmers in an individual farming site was cross-checked through FGD with farmers. From each of the farming sites populated with WSSV experienced shrimp farmers (Khulna – 500, Bagerhat – 90 and Satkhira – 190), about 30% of farmers each from Khulna (150), Bagerhat (26) and Satkhira (57) in a total of 233 farmers, who experienced WSD in the past years (from 2010 to 2017), were sampled using a simple random sampling technique that made a robust dataset for statistical analyses. The total number of semi-intensive and extensive farms were 21 and 212, respectively (Fig. 1). The questionnaire survey was conducted applying google survey form in the android mobile phone during December/2017 to July/2018. Before the survey, the paper-based questionnaire was tested at the farmer level, edited and finalized. Then the questionnaire was transformed into google featured questionnaire (made available at https://goo.gl/forms/ckG1AIf9xMTxtPpf1) and then applied by the trained enumerators to conduct the survey. After the survey, the dataset was downloaded in the computer from the Google in CSV (comma-separated values) format and then converted to a MS Excel file.

## Declaration of Competing Interest

The authors declare that they have no known competing for financial interests or personal relationships which have, or could be perceived to have, influenced the work reported in this article.

## Appendix Supplementary materials

Image, application 1

Image, application 2
